# Silver Mixture and Nervous Sedatives

**Published:** 1862-12

**Authors:** Ira Hatch


### CHICAGO MEDICAL EXAMINER.

N. S. DAVIS, M.D., Editor.

VOL. III. DECEMBER, 1862. NO. 12.

### Original Contributions.

ARTICLE XXXIX.

### SILVER MIXTURE AND NERVOUS SEDATIVES.

By IRA HATCH, M.D.

Read before the Chicago Medical Society, November 14th, 1862.

The iodide of potassium unites with the nitrate of silver in the proportions of 41 of the former, to 43 of the latter, when dissolved in pure water.

The iodide of silver is precipitated in the form of beautiful pale yellow crystals, leaving the nitrate of potassa in solution. By adding more of the iodide of potassium, the precipitate becomes white, and is a per iodide of silver. The utmost extent to which the iodide of potassium will unite with the nitrate of silver I have not ascertained. It is evident that it unites in different proportions. My formula is as follows:—

℞. Argenti Nitratis, ........................grs. xvi.
Potassii Iodide, ........................ " xxiv.
Aquæ Destillatæ, .......................℥iv.

Misce. Dose:—One teaspoonful three times per day, after eating.—Shake well.

When unwashed, this mixture contains the iodide of silver in the form of a precipitate, and the nitrate of potassa in solution, and, possibly, a small proportion of the iodide of potassium uncombined. In very sensitive stomachs, this mixture may possibly produce slight disturbance: if so, pour off the fluid from the precipitate, and fill up the phial with pure water. If you wish to wash the precipitate, dissolve 16 grs. iod. potass. in an ounce of water, then add of the nit. silver till a dense yellow precipitate appears; let it settle, pour off the water, and add 4 oz. pure water, and 8 grs. iod. potass. This will seldom be necessary, as the quantity of nit. potass. in solution is inconsiderable. It will very seldom disturb the stomach.

When thus prepared, I have never known it disturb the stomach of the most delicate woman or child.

To those who are in the habit of using the nitrate of silver internally, I would recommend the use of this mixture in its place; I believe it to be a sedative, allaying irritation of the mucous membranes. In all cases where the caustic properties of the nitrate are objectionable, this preparation possesses important advantages. By many physicians, the sedative properties of the nitrate of silver are supposed to depend upon its causticity. This is undoubtedly a mistake. The sedative properties of the oxide have long been known and admitted. It is now pretty generally understood that the non-caustic preparations of silver act equally as sedatives with the nitrate.

This being admitted, we can conceive of but few cases where it would be advisable to introduce a caustic into the stomach. I am satisfied that many stomachs will not tolerate it for any great length of time. The silver mixture of which I am speaking, may be given with impunity for any length of time. As a sedative it is equal, and as an alterative it is far superior to the nitrate.

It combines the most valuable properties of both the silver and the iodine in a form which the most delicate stomach will always tolerate. It is particularly adapted to those gastric affections which are attended with increased sensibility and increased secretion.

There can be no doubt that certain medicines act upon certain membranes.

The particular surfaces upon which the preparations of silver act, besides the mucous membranes of the alimentary canal, are the mucous membranes of the organs of generation.

It is also equally clear, that the glands in the vicinity of an inflamed or diseased mucous membrane are often, perhaps we may say generally, affected either by sympathy, or by the absorption of impure secretions.

The swelling of the inguinal glands and testicles in gonorrhœa, and of the submaxillary and parotid glands in scarlatina and diphtheria, &c., are familiar examples.

If then we can combine in a single medicine a powerful sedative influence upon the mucous surface itself, together with an active alterative influence upon the glands, we have a valuable remedy. These powers are contained in this combination of silver and iodine, to a considerable extent. It is not liable to decomposition, or loss of strength, has no disagreeable taste, and never disturbs the stomach. It may be given in liberal doses, and continued for a longer time than any other preparation of either silver or iodine. Many delicate females, who are afflicted with leucorrhœa, will not tolerate the nitrate. These patients generally have sensitive, irritable, inflamed stomachs.

The caustic properties of the nitrate, when it is given in efficient doses, always aggravate the gastric affection, and cannot be continued long enough to benefit the patient. But this mixture, while it relieves the uterine irritation, removes the gastric disturbance. In gastralgia and pyrosis, accompanied with pain in the stomach, nausea, and vomiting of a thin transparent fluid, intensly acid, attended often with extreme thirst; the silver mixture is a valuable remedy.

In these cases, alkalies and absorbents, bismuth, &c., afford only a temporary relief. Delicate females, tormented at the same time with gastric and uterine irritation, with vomiting, loathing of food, sour eructations, constipation, flatulency, pain in the side and back, and leucorrhœa may always be encouraged to expect relief by this remedy. This medicine is equally efficacious in idiopathic gastric irritation, whether in males or females, adults or children.

In cases of extreme nervous debility, some bitter tonic should be added. With me the hydrastis canadensis is a favorite article. The best mode of preparation is infusion of the root in cold water. The bitter acts directly upon the mucous membranes, not only of the mouth and throat, but also of the stomach, the urethra, and the uterus.

I have never seen a case of nursing sore mouth that would not yield to the action of the silver mixture and the hydrastis; the medical properties of the hydrastis deserve further notice. I do not pretend to say that this combination of sedative, alterative, and tonic, all exerting a specific influence upon the mucous membranes, is better than any other combination of remedies. But this I will say: that in the experience of their use for fifteen years, they have seldom disappointed my expectations. All ordinary cases of simple leucorrhœa without ulceration, will yield to their use; and leucorrhœa, with non-malignant ulceration, with the application of some tonic astringent powder to the ulcerated surface, dry, will yield to these remedies much sooner, and with far less trouble to the patient, than by the application of caustics. The pulverized gum catechu, is one of the best preparations; it may be applied by moistening a sponge, and dipping it in the dry powder, and pressing it directly upon the diseased surface. Dry calomel is also a good application to be used occasionally.

The application of caustics to the neck of the uterus for simple abrasions of the mucous surface, I believe to be unnecessary and barbarous. The beneficial effects of the silver mixture may be owing in part, perhaps, to its alterative and tonic properties; but I think it is mainly due to its sedative influence "*determined*" to the mucous surfaces.

It seems to be no longer a question whether we have *arterial sedatives*, the primary action of which is upon the pulsations of the heart, enabling the practitioner to control the pulse without wasting the vital powers.

Certain articles have long been known to possess these powers, yet the most efficient remedies were not fully understood. The introduction of the veratrum viride into general use as an arterial sedative forms an important epoch in the history of medicine, inasmuch as it has revolutionized practice in inflammatory diseases.

We need *nervous sedatives*, to act directly upon the periphery of the sentient nerves. We have some such remedies, but the fact seems hardly to be recognized by the profession. A good deal of attention has of late years been paid to reflex action. In proportion as these investigations progress, will the demand for these sedatives increase. A multitude of morbid actions owe their origin to irritation of the mucous surfaces, which irritation is communicated to distant parts of the system through the medium of the nerves. I have had some personal experience, for the last few weeks, upon this subject. Some weeks ago I had a severe attack of diarrhœa: in fact, it might with more propriety be called cholera, supervening upon chronic diarrhœa. The extremities were cold, the pulse had disappeared at the wrist, the vomiting and spasms of the muscles of the extremities were most distressing, and a fatal collapse was seriously apprehended. During convalescence I was troubled for a long time with numbness of the feet and legs, and had a sense of insecurity when walking; it required all my resolution to venture up and down stairs. This partial anesthesia was accompanied by a strange sensation in the back and limbs, and a constant fear of convulsions. These sensations wore off gradually; but they return whenever the diarrhœa returns; as it has several times since my sickness.

I have no doubt that a large share of the convulsive diseases have their origin in the irritation of mucous surfaces, especially among children. The convulsions arising from bowel-complaints, from worms, and from indigestion, as well as from the eruptive fevers, when the eruption is too long coming to the surface, and the force of the disease is spent upon the mucous membranes, are familiar examples. Now, what we want in these cases is a sedative, "determined to," and acting upon the primary points of irritation.

We are obliged to use narcotics, and thus partially suspend the functions of the brain and nervous centres, in order to allay the irritation at their extremities. Recently some physicians have substituted arterial sedatives for narcotics, it is said, with extraordinary success. Dr. NORWOOD strongly recommends the viratrum viride in convulsions. This is a movement in the right direction, and it is to be hoped will lead to the discovery of direct nervous sedatives. This is a subject that should engage the attention of the profession. These remedies should be sought for. No doubt there may be found those that will as effectually control the irritations of the mucous membranes, and thereby allay the sympathetic irritation of the brain and nervous centres, as our arterial sedatives now control the undue action of the heart and arteries; an operation but little understood a few years ago. As yet but little attention has been paid to this subject. I know of but one writer on materia medica that has attempted to classify these medicines. Those articles that are now in use, are weak and slow in their operation, and the power is hardly recognized by the profession. But no doubt there is such a power. If, "to be able to control *vascular* excitement, whenever it occurs, either when it is a curative effort of nature, passing salutary bounds, or itself a diseased action, be a most valuable acquisition," certainly it can be no less so to be able to control *nervous* excitement. Mercury is a nervous sedative in the sense in which I use the term.

No one can doubt that it possesses this power, who has watched its effects in small doses, in cholera infantum, or in larger doses, in cases of convulsions in children. Its effect is often so prompt and immediate that we can hardly attribute it to any other power but that of a direct sedative. The different preparations of silver possess this power to a considerable extent. This is well understood by surgeons, when they use it topically in irritable mucous membranes, as the eye, the urethra, &c. In certain irritable stomachs, this power is well marked; it not only acts as a direct sedative upon the mucous membrane of the alimentary canal, but upon the mucous membranes of the urinary organs, and the organs of generation.

Lead is a well-known nervous sedative, whether used as a local or as a constitutional remedy. Zinc possesses the same power, and through this power cures epilepsy and chorea, depending upon irritation of the extremities of the nerves.

Muriate of ammonia, iodide of potassium, chlorate of potassa, alum, bismuth, &c., all possess this same power; some operating upon one set of membranes, and others upon another set of membranes. Whether any combination of these medicines, or of others not mentioned, would increase the effect, is a question worthy of consideration.

I am satisfied that the iodide of silver is a more active nervous sedative than either silver or iodine separately. This leads me to conclude that other combinations may be found far more active than any yet discovered. And we have reason to hope that a remedy will yet be found to control the irritation of every distinct mucous membrane in the body. We have stimulants and alteratives that act directly upon certain membranes.

No one can doubt that copaiba, cubebs, buchu, gallium, aperine, hydrastis canadensis, uva ursi, matico, and other articles act directly upon the mucous membranes of the urinary organs. And no one can doubt that the power of these articles is increased by combination. The best preparation I have seen, is by Mr. WILLARD, a druggist in this city, prepared in the shape of lozenges, under the name of French Tablets.

We have other tonics and stimulants that act upon the mucous membranes of the respiratory passages, as the squill, &c., and others still upon the mucous membranes of the alimentary canal. All these are well understood. But in sedatives to allay irritation of the nervous extremities, without disturbing the functions of the brain and nervous centres, we are deficient. *Hic labor hoc opus est.*

